# A Newborn with Cleft Palate Associated with PTEN Hamartoma Tumor Syndrome

**DOI:** 10.3390/clinpract15010022

**Published:** 2025-01-20

**Authors:** Ulf Nestler, Daniel Gräfe, Vincent Strehlow, Robin-Tobias Jauss, Andreas Merkenschlager, Annika Schönfeld, Florian Wilhelmy

**Affiliations:** 1 Department of Neurosurgery, University Hospital, 04103 Leipzig, Germany; florian.wilhelmy@medizin.uni-leipzig.de; 2 Institute for Pediatric Radiology, University Hospital, 04103 Leipzig, Germany; daniel.graefe@medizin.uni-leipzig.de; 3 Institute of Human Genetics, University of Leipzig Medical Center, 04103 Leipzig, Germany; vincent.strehlow@medizin.uni-leipzig.de (V.S.); robin-tobias.jauss@medizin.uni-leipzig.de (R.-T.J.); 4 Division of Neuropediatrics, University Hospital, 04103 Leipzig, Germany; andreas.merkenschlager@medizin.uni-leipzig.de; 5 Department of Oral and Maxillofacial Surgery, University Hospital, 04103 Leipzig, Germany; annika.schoenfeld@medizin.uni-leipzig.de

**Keywords:** development delay, macrocephaly, subcutaneous lipoma, palate cleft, pediatric, PTEN, hamartoma tumor syndrome

## Abstract

**Background**: PTEN hamartoma tumor syndrome (PHTS) has evolved into an umbrella term for a range of syndromes, characterized by loss-of-function variants in the phosphatase and tensin homolog (PTEN) tumor suppressor gene on chromosome 10q23.31. This can result in a lifelong tumor predisposition in patients. Often, the syndrome is diagnosed in early childhood because of macrocephaly, dermatological findings, or development delay. Since the correlation between phenotype and genotype is weak, and the penetrance is age-dependent, this poses the question of the appropriate timing of potentially invasive and burdensome examinations for early cancer detection. **Case**: The present report describes an infant with cleft palate associated with PHTS, a rare occurrence, though the initial report of Cowden syndrome already pointed to oromaxillofacial abnormalities. The recent pediatric literature is reviewed to assess which clinical symptoms should raise suspicion of PHTS and may then lead to early genetic counseling. **Conclusion**: Since the amount of prospective data remains limited, and the estimation of tumor risk during infancy and adulthood is very difficult, we advocate for early and broad genetic testing in suspected cases, to gain more insights into this rare disease and allow for better counseling for patients and their families.

## 1. Introduction

PTEN hamartoma tumor syndrome (PHTS) encompasses a range of syndromes, including Cowden syndrome, Bannayan–Riley–Ruvalcaba syndrome and Lhermitte–Duclos disease. These syndromes are caused by loss-of-function and null variants in the phosphatase and tensin homolog (PTEN) tumor suppressor gene on chromosome 10q23.31, leading to a lifelong predisposition to tumor development [1]. The genotype-phenotype correlation is weak, the penetrance is age-dependent, and the description of the phenotype expression is still evolving [2,3].

The initial description of Cowden syndrome included orofacial dysmorphism, and facial and oral examinations can reveal aberrations in about one third of cases [4,5]. Recent pediatric publications have primarily focused on skin stigmata, macrocephaly, and developmental delay, partly influenced by the subspecialty of the initial examining physician (Table 1). Here, we report on the phenotypic spectrum in a case of cleft palate associated with PHTS in a newborn, a coincidence that has not been reported before.

Cleft palate occurs in about 1 to 2 of 1000 live births, with, in recent years increasing antenatal ultrasonographic detection rates. An association with chromosomal aberrations is only found in about 10% of these cases, without a suggested link with a specific syndrome [6].

The secondary palate is formed by the palate shelves growing out from the maxillary processes, orientating vertically and upward from the tongue around the eighth embryonal week. Fusing and elevation start in the anterior third of the palate, progressing from there during the ninth week. By week ten, mesenchymal differentiation into the hard and soft palate is complete [7]. Though a multitude of genes is involved in these processes, a specific link to PTEN activity has not been found in cleft palate. Theoretically, downstream from PTEN-altered PI3K signaling, pathway cross-talk modifications may be postulated, e.g., on Forkhead Foxf1 or Gsk3beta, which have been shown to be involved during palate forming [8]. The data presented here, has been published as a preprint [9].

**Table 1:** Childhood PHTS symptoms reported over the last ten years, listed according to the number of patients examined for each specific symptom. Though the pediatric focus on reporting PHTS symptoms was found to be on cutaneous stigmata, macrocephaly, and cardiovascular changes, the most frequent symptoms were macrocephaly, gastrointestinal polyposis, and developmental delay.

Year	2024	2024	2020	2019	2019	2019	2018	2017	2015	2015	∑		2024
author	Bregvadze [10]	Martín-Valbuena [11]	Martin [5]	Ciaccio [12]	Plamper [13]	Yotsumoto [14]	Kato [15]	Hansen-Kiss [16]	Busa [17]	Smpokou [18]	cases	percentage	present case
cases	1	11	13	16	23	1	6	47	7	34	159		
male	1	7	13	14	15	1	2	29	3	23	108 out of 159	68%	male
skin observations *	1 out of 1	3 out of 10	13 out of 13	7 out of 16	17 out of 23	1 out of 1	1 out of 6	30 from 47	4 out of 7	12 out of 31	89 out of 155	57%	nuchal naevus flammeus
macrocephaly	1 out of 1	7 out of 11	13 out of 13	16 out of 16	23 out of 23	1 out of 1	6 out of 6	46 from 47	6 out of 7	27 out of 27	146 out of 152	96%	yes
cardic/vascular		0 out of 11		3 out of 16	1 out of 23			1 from 47	1 out of 7	16 out of 34	22 out of 138	16%	persistent ductus arteriosus
developmental delay	1 out of 1	8 out of 11	9 out of 13	9 out of 16	10 out of 23	1 out of 1	6 out of 6	15 from 33	4 out of 7	23 out of 25	86 out of 136	63%	motor
malignancy observed **		0 out of 11	1 out of 13	0 out of 16	2 out of 7			0 out of 47		5 out of 34	8 out of 128	6%	no
thyroid abnormalities ***	1 out of 1	1 out of 11	4 out of 13	1 out of 16	14 out of 23			7 out of 27		10 out of 18	38 out of 109	35%	no
autism spectrum disorder	1 out of 1	3 out of 11		4 out of 16	1 out of 23	1 out of 1	1 out of 6	25 out of 33	1 out of 7	7 out of 7	44 out of 105	42%	n.a
genital lentiginosis	1 out of 1	0 out of 11	6 out of 6	0 out of 14	8 out of 15			12 out of 29	0 out of 3	19 out of 19	46 out of 98	47%	no
facial dysmorphism		5 out of 11		14 out of 14		1 out of 1	6 out of 6	1 out of 47			27 out of 79	34%	no
gastrointestinal polyposis	1 out of 1				3 out of 3			6 out of 10		9 out of 12	19 out of 26	73%	n.a.
overgrowth	1 out of 1	3 out of 11							3 out of 7		7 out of 19	37%	no
oral mucosal papillomatosis			4 out of 13								4 out of 13	31%	no
oral dysmorphism		3 out of 11									3 out of 11	27%	cleft palate
MRI white matter hyperdensity		1 out of 7		4 out of 16	7 out of 15	0 out of 1	2 out of 6	5 out of 16	3 out of 5		22 out of 66	33%	yes
MRI cerebellar signs		2 out of 7		6 out of 16	1 out of 15	0 out of 1		0 out of 16			9 out of 55	16%	no
MRI enlarged perivascular spaces		2 out of 7		10 out of 16	3 out of 15	1 out of 1	1 out of 6		3 out of 5		20 out of 50	40%	yes

* lipoma/hemangioma/hamartoma/café-au-lait spots/trichilemmoma; ** 7 cases of thyroid cancer, one renal cell carcinoma; *** nodules, goiter, carcinoma; n.a.: not assessed.

## 2. Case Description

The male infant was born by planned cesarean section at 38 weeks of gestation due to polyhydramnios and macrocephaly. His birth weight was 3972 g (1.5 z), his length was 52 cm (0.4 z), and his head circumference was 40 cm (3.6 z).

**Figure 1:**
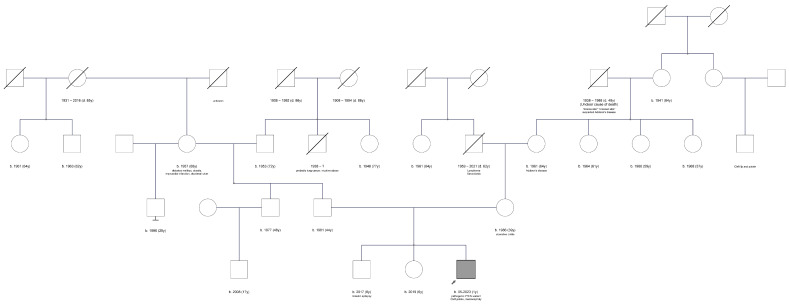
Pedigree of the family obtained during genetic counseling.

The patient is the third child of non-consanguineous parents. His 6-year-old brother had suffered from a single epileptic seizure with suspected self-limited focal epilepsy of childhood (“Rolandic”). The 4-year-old sister and the father are healthy, while the mother has ulcerative colitis, and a cousin of the maternal grandmother had a cleft palate and lip (Figure 1).

Postnatal respiratory adaptation was prolonged and complicated by hypoglycemic episodes. A cleft soft palate was detected shortly after birth, and, together with macrocephaly, prompted genetic counseling.

Genetic testing was performed due to the heterogeneous symptoms of the patient (e.g., macrocephaly, cleft palate). All protein-coding genes were assessed (i.e., whole-exome sequencing (WES)) with a specific emphasis on genes with a known disease association. Variants were filtered based on impact on protein structure, prevalence in the general population, and evidence in the literature. Whole-exome sequencing with subsequent segregation analysis of the parents revealed a heterozygous, de novo, null variant c.184A>T, p.(Lys62*) in the *PTEN* gene. The variant has previously been reported as pathogenic (ClinVar-ID: 1069915).

The transcript NM_000314.8 was used. The variant was classified as pathogenic based on the following ACMG criteria: PVS1, PS2_MOD, PS4_SUP, and PM2. The variant has been submitted to ClinVar twice, with one submission being this specific case (SCV004812067). The variant has hitherto not been reported in the literature. However, reports of other loss-of-function/null variants have been published.

**Figure 2:**
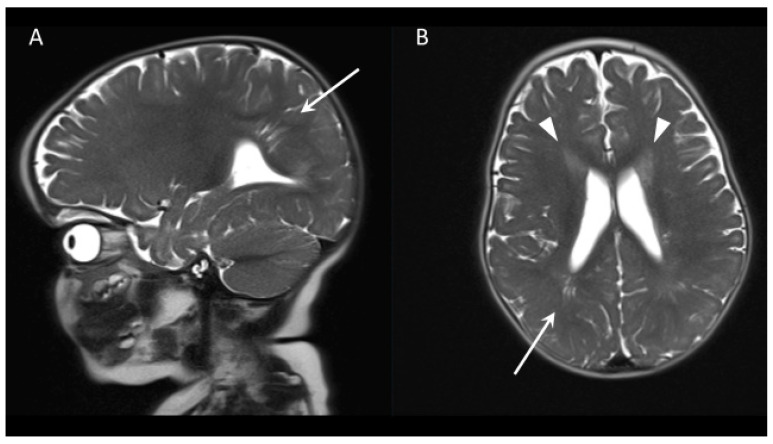
Cranial MRI at 10 months of age. T2-weighted fast spin-echo sequence in (**A**) sagittal and (**B**) axial orientation. Right occipital stria-like enlarged Virchow–Robin spaces (arrow). T2-hyperdensities rostral to the ventricles (arrowheads).

The child was referred to an oromaxillofacial surgeon, who scheduled surgical closure at 10 months and initiated magnetic resonance imaging (MRI) to rule out hydrocephalus before surgery (Figure 2). Examination at 10 months revealed brown skin patches on both knees and soles, without penile freckling. The head circumference was 50 cm (2.8 z), and no partial overgrowth was observed. A persistent foramen ovale and ductus arteriosus, and mild mitral insufficiency were noted. Despite starting to roll over at 6 months of age, the boy was showing mild motor development delay, not yet being able to sit. A detailed diagnostic work-up for PHTS-related symptoms, including thyroid abnormalities, gastrointestinal polyposis, and autism spectrum disorder, was planned according to German guidelines [19].

## 3. Discussion

PTEN hamartoma tumor syndrome presents a diagnostic challenge. On one hand, patients have a tumor predisposition, with a 6% risk of developing a malignant tumor in childhood. On the other hand, screening for cancer or benign neoplasms in young children poses additional risks due to repeated anesthesia or invasive procedures. Thus, the appropriate timing of routine, guideline-driven diagnostic evaluations remains a matter of debate [18].

Macrocephaly is often an obvious symptom and can be detected during prenatal obstetric ultrasound examinations, whereas, in our case, this was additionally complicated by polyhydramnios. Routine sonographic assessment should be scheduled every 10th week of gestation. From the second trimester onward, fetal MRI can help in detailing anatomic structures when ultrasonography remains equivocal. After birth, transfontanellar ultrasonography and cerebral MRI are indicated to rule out the indication for a neurosurgical intervention, such as in hemorrhage, hydrocephalus, or tumors.

Although MRI findings are characteristic and present in up to 40% of patients, they are not specific to PHTS (Figure 2) [20]. Hydrocephalus in the setting of cranial dysmorphism in pediatric PHTS can result from Chiari malformation or arise as secondary to dysplastic cerebellar gangliocytoma in Lhermitte–Duclos disease. However, in the review of the 159 patients in Table 1, only one ventriculo-peritoneal shunt procedure for pseudotumor cerebri was reported (0.6%) [13].

Skin stigmata, such as lipoma, hemangioma or hamartoma, trichilemmoma, and café au lait spots are detectable by inspection. These signs are generally well documented; in the literature reviewed for Table 1, examination results are given for 155 out of 159 patients. The occurrence of subcutaneous lipoma, which is a rare finding in children, particularly when combined with macrocephaly, should raise suspicion of PHTS and prompt genetic testing [5].

Reports of PTEN-associated cleft palate are scarce, although orofacial dysmorphism and a high-arched palate are present in 27% to 34% of cases, according to the references included in Table 1. A single case of bifid uvula was described in 2007 [14]. Considering that up to 31% of cases present with oral mucosal papillomatosis and that oral hyperkeratosis has been described as part of Cowden syndrome, oral examination is an important adjunct in diagnosing PHTS [21]. However, our overview suggests that oral signs are less frequently described in the diagnostic process compared to the more prominent symptoms, with oral items reported in only 24 cases from 159 observations (Table 1).

It remains elusive whether this case represents a causal or casual coincidence. A genetic link between our patient’s cleft palate and that of the maternal grandmother’s cousin is unlikely due to the distant relationship and the frequency of cleft palates in the general population (1–2:1000). Given the clinical symptoms and standardized variant classification, the identified variant in *PTEN* was the only variant of clinical significance. It is absent from the general population, and its type is a stop-gain variant, which means that no functional protein product is translated (i.e., null variant).

The development of cleft palate is complex and multifactorial. There are a number of genetic diseases with an increased risk, such as 22q11.2 deletion syndrome, Stickler syndrome, or Beckwith–Wiedemann syndrome. PTEN is a major lipid phosphatase that downregulates the PI3K/AKT pathway to cause G1 cell cycle arrest and apoptosis. When PTEN is absent, decreased, or dysfunctional, the phosphorylation of AKT1 is uninhibited, leading to the inability to activate cell cycle arrest and/or to undergo apoptosis.

In addition, through a lack of protein phosphatase activity, the mitogen-activated protein kinase (MAPK) pathway is dysregulated, leading to abnormal cell survival [22]. This dysregulation could have an influence on the connection of the palatal arches and thus could have contributed to the development of cleft palate. Since this is the first report on the association of a cleft palate with a *PTEN* mutation, an estimation of the resulting tumor risk or phenotype expression pattern cannot be derived from the literature.

In the present case, genetic testing not only identified the cause of the existing abnormalities but also resulted in recommendations for follow-up examinations, particularly with regard to the increased risk of tumors in the patient (see https://www.ncbi.nlm.nih.gov/books/NBK1488/#phts.Management, accessed on 9 January 2025). By testing the parents, a statement could be made about the risk of PTEN disease in further children. As the variant is probably new (de novo), the risk of recurrence is low (<1%). However, a germ cell mosaic cannot be ruled out. The clinical picture, inheritance, the risk of recurrence, and follow-up examinations were explained to the parents during genetic counseling.

Data collection and analysis in PHTS are limited by the retrospective nature of most case reports and reviews. The data do not allow us to predict tumor risk during infancy or to estimate how many patients live with a pathogenic variant in *PTEN* without ever requiring medical attention. We advocate for broad genetic testing like WES with a low threshold of suspicion in order to anticipate developmental delays, potentially detect tumor growth at an early stage, and support patients and families in managing this rare, burdensome disease.

## Data Availability

The original contributions presented in this study are included in the article material. Further inquiries can be directed to the corresponding author.
